# What Is the Effect of Topiramate Use on Growth in Children With Epilepsy?

**DOI:** 10.7759/cureus.28503

**Published:** 2022-08-28

**Authors:** Muhammad T Alrifai, Noura A Alsubaie, Albatool M Abodarahem, Shadan B Alomran, Maryam N Alboqami, Raghad T Alsadun, Yusra Sajid Chachar, Mohammed A Alqassim, Mohamed K Abdelkabir

**Affiliations:** 1 Pediatrics Department, Neurology Division, King Abdullah Specialized Children's Hospital, King Abdulaziz Medical City, Riyadh, SAU; 2 Pediatrics Department, King Saud Bin Abdulaziz University for Health Sciences College of Medicine, Riyadh, SAU; 3 Pediatrics Department, King Abdullah International Medical Research Center, Riyadh, SAU; 4 Biostatistics and Epidemiology Department, King Saud Bin Abdulaziz University for Health Sciences College of Medicine, Riyadh, SAU

**Keywords:** epilepsy, weight, topiramate, kingdom of saudi arabia (ksa), body mass index: bmi

## Abstract

Objective

The literature related to weight loss as a side effect of using topiramate (TPM) in pediatric patients is inconsistent. The aim of this study was to assess the effect of TPM on the growth of pediatric epileptic patients.

Methods

The electronic medical files of 50 pediatric epileptic patients who were prescribed TPM over 5 years were retrospectively reviewed. Cases treated with other antiepileptic drugs were the control group (n=60).

Results

Height growth was similar in both groups. At the 6-12-month follow-up, there was a decrease in the average BMI in the TPM group of -0.81 kg/m2 (p=0.019) and an increase in the control group of +0.46 kg/m2 (p=0.023). Weight loss was noted in 21/50 (42%) of the TPM group as compared with 13/60 (22%) in the control group (p=0.02). More weight loss was observed in the overweight TPM group in 7/16 (44%) compared to none in the nine cases in the control group (p=0.03). After the one-year follow-up, the average change in weight was +1.73 kg (p=0.0001) and +3.53 kg (p=0.0001) in the TPM and control groups, respectively. In patients with normal initial BMI, the weight increased by +1.3 kg on average, compared to the group with a high initial BMI, which decreased by -2.55 kg.

Conclusion

Topiramate use has no negative effect on height growth in pediatric patients with epilepsy. While mild weight loss occurs frequently in the first year of treatment, weight gain resumes after the first year except in patients with a high initial BMI

## Introduction

Epilepsy is a chronic neurological disorder that manifests in recurrent unprovoked seizures [1]. It is one of the most prevalent neurological disorders in children, with an incidence rate of 58-144 per 100,000 person-years and a prevalence of 0.5-1% [2]. Topiramate (TPM) is an effective drug used to treat epilepsy in children, both as monotherapy [3] and as add-on polytherapy [4,5]. Several mechanisms of action of TPM have been proposed, including sodium channel inactivation, inhibition of glutamate activity, and enhancement of γ-Aminobutyric acid (GABA) activity [6]. It has been approved as effective in treating children older than 4 years with refractory partial-onset seizures, Lennox-Gastuat syndrome, and generalized tonic-clonic seizures [7]. Other studies showed a positive effect on migraine in children [8-10], which led to approval by the Food and Drug Administration (FDA) for this indication; however, conflicting results were later reported [11].

The side effects of TPM vary from patient to patient. The most frequently reported side effects are poor appetite, abdominal pain, difficulties in concentration, sedation, weight loss, and paresthesia [3-5,8,11-13]. The side effects related to TPM in randomized trials were mild to moderate and frequently temporary, and infrequently (5%), they caused discontinuation of treatment [7]. Weight loss due to TPM therapy was reported in adult studies, ranging from 17%-86% [14-17]. Similarly, weight loss in children was reported with variable rates of 0%-81% [3,5,8,18,19]. The mechanism of weight loss due to TPM treatment remains unclear. Potential mechanisms causing weight loss include an effect on hypothalamic neuropeptides, glutamate antagonism, and insulin sensitivity [20]. Literature reporting weight loss and decrease of BMI in terms of the duration of TPM therapy varied. A study reported weight loss in the first 6-9 months, but weight gain resumed afterward [19], while another study reported weight loss in the first 12-18 months of treatment and weight gain thereafter [21]. In another study, a decrease in BMI was noted at 12 and 24 months, but not at 36 months of TPM treatment [22].

The aim of this study was to assess the effect of TPM on the height, weight, and body mass index (BMI) of pediatric epileptic patients who were attending a tertiary center in Riyadh, Saudi Arabia.

## Materials and methods

This retrospective cohort study was conducted from 1 January 2015 to 31 August 2020 at King Abdullah Specialized Children's Hospital (KASCH), Riyadh, Saudi Arabia. KASCH is a 350-bed specialized children’s hospital that is part of King Abdulaziz Medical City (KAMC), Riyadh, Saudi Arabia. The data were abstracted from the patients’ electronic medical records using BESTCare 2.0A (ezCaretech, South Korea).

Study participants

The records of Saudi pediatric epileptic patients who were prescribed TPM and who were aged from 1 month to 18 years in the study period were included. The control group included all pediatric epileptic patients who were prescribed any other antiepileptic therapy. The data for both groups were collected from the time of the highest dose of medication and used for a minimum of 6 months. The first follow-up was at 6 to 12 months (P1), and, if available, a follow-up after 12 months was considered the second follow-up (P2). If the patient was seen more than once in a period, the first visit was taken for analysis.

Patients who were taking medications known to affect growth in children such as valproic acid, zonisamide, vigabatrin, and stimulants were excluded. Furthermore, exclusion criteria included patients with disorders that affect growth in children like gastrointestinal disorders, gastrostomy/nasogastric tube feeding, systemic illnesses, eating disorders, and significant psychiatric disorders.

The data were collected retrospectively and included demographic data, specifically age and gender. In addition, data regarding the seizure type, antiepileptic drugs, and response to treatment and comorbid disorders were collected.

Weight and height were measured using the Seca scales at the pediatric neurology clinics. The assessments were obtained by trained personnel in a standardized manner using the same methods of measurements using calibrated instruments. The variables included age in years, gender, weight in kilograms (kg), and height in centimeters (cm). BMI was calculated by the metric formula, dividing the weight in kilograms by the height in square meters. BMI was categorized according to the Centers for Disease Control and Prevention’s (CDC's) criteria with age-percentile growth charts. BMI of lower than 5% was considered underweight, 5%-85% as healthy weight, 85%-95% as at risk of overweight, and more than 95% as overweight. The growth parameters were recorded at P1 and P2. Weight loss was defined as any decrease of weight at follow-up below the initial weight. Significant weight loss was defined as when the weight loss is 5% or more of the initial body weight.

Data analysis

The data were analyzed using SPSS version 16 (SPSS Inc., Chicago, IL). The data are presented as the mean and standard deviation (SD) for continuous variables and frequency and percentage for the categorical variables. Statistical analysis tests for both continuous and categorical variables were used for the TPM group versus the control group. The paired samples t-test was used to determine the effect of TPM and other antiepileptic drug exposure on changes in the growth parameters in P1 and P2. A p-value of less than 0.05 was considered statistically significant.

Institutional review board approval was obtained from King Abdullah International Medical Research Center, number RYD-19-41981257630.

## Results

In total, 110 patients were eligible for the study, with 55% (n=60) being female and the mean age being 7.1±039 years with a range of 1.16-18 years. The TPM group consisted of 50 patients, and 60 were in the control group. Table 1 shows no difference between the two groups regarding demographic information and comorbid developmental delay and cerebral palsy. The two groups were matched in terms of monotherapy versus polytherapy; however, the TPM group had more cases of intractable epilepsy.

**Table 1:** Compression of the TPM vs non-TPM groups with regards to demographics, epilepsy therapy, and comorbidities TPM: topiramate

	TPM (n=50)	Non-TPM (n=60)	P-value
Gender (M)	23 (46%)	27 (45%)	0.91
Age (yr) mean±SD	8.09±5.46	3.34±3.99	0.07
Age median (yr)	7	6	
Developmental delay	14 (28%)	11 (18%)	0.22
Cerebral palsy	3 (6%)	2 (3%)	0.65
Monotherapy	13 (26%)	12 (20%)	0.45
Polytherapy	37 (74%)	48 (80%)	
Controlled	18 (36%)	35 (58%)	0.02
Intractable	32 (64%)	25 (42%)	

The mean initial BMI was 18.5±10.5 in the TPM group and 15.7±3.1 in the control group (p=0.005), with the median initial BMI of 15.7 and 15.5, respectively. The TPM group had a higher proportion (16/50, 32%) of patients with an initial high BMI compared to the control group (9/60, 15%) (p=0.03) (Table 2).

**Table 2:** Compression of the TPM and non-TPM groups regarding weight loss at P1 (6-12 months) and P2 (after 12 months) BMI: body mass index; wt: weight; TPM: topiramate

	TPM (n=50)	Non-TPM (n=60)	P-value
Initial BMI (mean±SD)	18.5±10.5	15.7± 3.1	0.00
Initial high BMI	16(32%)	9(15%)	0.03
Wt loss at P1	21(42%)	13(22%)	0.02
Significant wt loss at P1	5/50(10%)	4/60(7%)	0.73
Wt loss in overweight at P1	7/16(44%)	0/9(0%)	0.03
Follow up at P2 (n)	30	48	
Wt loss at P2	5/30(17%)	4/48(8%)	0.30
Significant Wt loss at P2	2/30(0.1%)	0/48(0%)	0.14
Wt loss in overweight at P2	3/10(30%)	0/6(0%)	0.25

Growth changes during P1 (6-12 months)

In P1, the average increase in height was +2.5 cm in both groups, and the average increase in weight was +0.64 and +1.51 kg in the TPM and control groups, respectively (Table 3). However, there was an average decrease in the BMI in the TPM group of -0.81 kg/m2 (p=0.019) and an increase in the control group of +0.46 kg/m2 (p=0.023) (Table 3). The normal and underweight subgroups of the BMI (n=34) showed a reduction of -0.83 kg/m2 from an average of 13.92 to 13.09 kg/m2, and the overweight and the at-risk-of overweight group (n=16) decreased by -0.81 kg/m2 from an average of 28.29 to 27.48 kg/m2. The control group, irrespective of the initial BMI, gained statistically significant height and weight, but the BMI remained unchanged, with a marginal increase of +0.69 kg/m2 in the normal/underweight subgroup (n=52) and +0.78 kg/m2 in the overweight and at risk of overweight subgroup (n=9) (Table 3).

**Table 3:** Compression of the TPM and non-TPM groups regarding height, weight, and BMI changes at P1 (6-12 months) and P2 (after 12 months) Nl: normal; BMI: body mass index; Wt: weight; TPM: topiramate

	TPM (n=50)	Non-TPM (n=60)	P-value
Initial BMI (mean±SD)	18.5±10.5	15.7± 3.1	0.00
Initial BMI (median)	15.7	15.5	
Initial high BMI	16 (32%)	9 (15%)	0.03
P1 period	TPM (n=50)	Non-TPM (n=60)	
Change of height (cm)	+2.5	+2.5	
Change of weight (kg)	+0.64	+1.51	
Change of BMI (kg/m^2^)	-0.81	+0.46	
Nl/underweight subgroup change BMI (kg/m^2^)	-0.81	+0.69	
Overweight subgroup change BMI (kg/m^2^)	-0.83	+0.78	
P2 period	TPM (n=30)	Non-TPM (n=48)	
Change of height (cm)	+4	+5	
Change of weight (kg)	+1.73	+3.53	
Change of BMI (kg/m^2^)	+0.33	+0.96	
Nl/underweight subgroup change wt (kg)	+1.37	+1.99	
Overweight subgroup change wt (kg)	-2.55	+3.3	

In P1, 21/50 (42%) of the TPM group lost weight as compared with 13/60 (22%) in the control group (p=0.02) (Table 2). However, significant weight loss (% of body weight) occurred in only five and four cases of the TPM and control groups, respectively. A higher proportion (7/16, 44%) lost weight in the overweight TPM group as compared to 0/9 in the control group (p=0.03).

Growth changes during P2 (>12 months)

In P2, the TPM group had 30 participants and the control group had 48. The average change in height was +4 cm (p=0.0001) and +5 cm (p=0.0001) in the TPM and control groups, and the average change in weight was +1.73 kg (p=0.0001) and +3.53 kg (p=0.0001), respectively. The average change in BMI was +0.33 kg/m2 in the TPM group (p=0.37) compared to +0.96 kg/m2 in the control group (p=0.007) (Table 3).

The interaction of TPM status and the initial BMI category indicated that irrespective of their status, all children gained significant height. The initial binomial BMI category in the TPM group had no significant changes in the BMI of the subgroups (normal/underweight and overweight/at risk of overweight), p=0.420 and p=0.576, respectively. The weight of the overweight and at-risk-of overweight subgroup decreased significantly by -2.55 kg from an average of 54.54 to 51.99 kg (p=0.048); however, the weight of the normal and underweight subgroup increased significantly by +1.3 kg, from an average of 15.86 to 17.23 kg (p<0.0003). In the control group, the normal and underweight subgroups had a significant increase in both weight and BMI (p=0.0001) and (p=0.010), respectively.

In P2, weight loss occurred in 5/30 (17%) of the TPM group compared with 4/48 (8%) in the control group (p=0.3) (Table 2). Weight loss of 5% or more of body weight occurred in only two cases of the TPM group and in none in the control group. Weight loss occurred in 3/10 in the TPM group with a high initial BMI as compared with 0/6 in the control group (p=0.25).

## Discussion

In the current study, TPM treatment in a group of Saudi pediatric patients with epilepsy did not affect height growth, but weight loss was prevalent, specifically in the first 6-12 months of treatment. Weight gain resumed after 12 months of treatment. Weight loss was more prominent in patients with a high initial BMI.

Lee et al. compared a control group without any medication after one year of valproic acid (VPA) treatment in a group of epilepsy patients and reported a decrease in height gain, with no effect on height growth in the group treated with TPM [23]. VPA, in contrast to TPM, caused a decrease in the proliferation of growth plate chondrocytes. A similar finding of the reassuring effect of TPM treatment on height growth was seen in our study.

A study assessing the effect of VPA and TPM on weight growth in pediatric epilepsy patients observed significant weight gain in patients treated with VPA (n=25), with no weight change at the 6-month follow-up in the TPM group (n=23) [18]. In a study with 80 patients with primary generalized seizures who were enrolled in a randomized controlled trial (RTC) for 20 weeks, 15% of the TPM group sustained weight loss compared with 2% in the control group [5]. In another RCT of 151 pediatric patients with epilepsy, weight loss was observed in about 35% at the last follow-up visit of patients being treated with TPM [3]. In another open study of 39 pediatric epilepsy patients treated with TPM for at least 9 months, weight loss was observed in 39% of the cases [19]. In an RCT with 44 patients treated for migraine and followed for 4 months, 81% of the TPM group lost weight compared with 14% in the control group [8]. In the current study, 42% in the TPM group sustained weight loss in P1 compared with 22% in the control group, but the weight loss was minor in most cases. Only five cases had a significant weight loss of ≥ 5% of the initial body weight as compared to four in the control group. The reasons for such variability in the rate of weight loss with TPM therapy remain unclear, but the small sample size in some studies and the short duration of therapy in others may explain such variability.

There is a variable effect of the duration of TPM treatment on weight loss. Uldall et al. reported weight loss in the first 6-9 months in their cohort of 39 pediatric epilepsy patients, but weight gain resumed afterward [19]. Rieter et al. conducted a study in which the duration of treatment was 36 months. The cohort was 53 children with epilepsy. There was a statistically significant decrease in BMI at 12 and 24 months but not at 36 months. The majority of the patients (90%) were on polytherapy, with 65% on valproic acid or vigabatrin [22]. In a group of 22 adult patients with epilepsy, weight loss was observed in the first 6 months in 59%, with weight gain resuming afterward [17]. In the current study, weight loss was observed in the first 12 months of treatment, and weight gain resumed afterward, except for the group with an initial high BMI who continued to lose weight, a desirable side effect in this subgroup of patients. The reasons for this variability are unclear. The small sample size of the groups at the P2 follow-up in our study and other studies may have contributed to these results [17,19,22]. Other medications that may affect weight such as valproic acid and vigabatrin, which were used in addition to TPM in some studies, may also have contributed to this finding [22].

BMI is a reliable, simple measure to predict weight growth in children [24]. In a study assessing the effect of treatment with TPM on weight in children, a higher initial BMI predicted weight loss at follow-up [22]. At the 24 and 36-month follow-ups, the BMI decreased more in patients with a higher baseline BMI [22]. The current study had a similar finding as patients with a higher initial BMI continued to lose weight after one year of treatment, in contrast to patients with a normal or low initial BMI. In a prospective study investigating the effect of TPM on weight loss in adults with epilepsy who were followed for one year, weight loss was also more prominent in patients with a high initial BMI [16]. The effect was due to an effect on appetite, and the weight loss was related mainly to fat loss rather than lean body weight, which may explain the increased effect on patients with a higher initial BMI.

The retrospective nature of our study causes some limitations that are inherent in this type of study. Another limitation is the sample size, which was relatively small, although larger than samples in several other reports. Despite these limitations, our study is the first to evaluate the effect of TPM in pediatric epileptic patients on all growth parameters, including height, weight, and BMI. It is reassuring that TPM has no negative effect on height growth. Furthermore, our findings add insight into the variability of the effects of TPM on weight growth in a pediatric age group, which may dictate the need for prospective studies with larger sample sizes and long-term follow-up with TPM as monotherapy to clarify such effects including the dosing effect.

## Conclusions

Topiramate use has no negative effect on height growth in pediatric patients with epilepsy. Mild weight loss is prevalent in the first year of treatment with topiramate in children with epilepsy. However, upon further follow-up, weight gain tends to resume afterward in children with normal or low initial BMI while weight loss continues after the first year in patients with a high initial BMI, making such an effect desirable in this subgroup of patients. Patients treated with topiramate tend to have more intractable epilepsy and the effect of other antiepileptic drugs on weight remains likely. Further prospective studies with monotherapy for long periods are needed to sort out the true effect of this drug on weight.
